# Study of the Temperature- and Pressure-Dependent Structural Properties of Alkali Hydrido-*closo*-borate Compounds

**DOI:** 10.1021/acs.inorgchem.1c03681

**Published:** 2022-03-24

**Authors:** Romain Moury, Zbigniew Łodziana, Arndt Remhof, Léo Duchêne, Elsa Roedern, Angelina Gigante, Hans Hagemann

**Affiliations:** † Department of Physical Chemistry, University of Geneva, 30 Quai E. Ansermet, Geneva 1211, Switzerland; ‡ Institut des Molécules et Matériaux du Mans, University of le Mans, Avenue Olivier Messiaen, Le Mans 72085, France; § Institute of Nuclear Physics, Polish Academy of Sciences, ul. Radzikowskiego 152, Kraków 31342, Poland; ∥ Empa, Swiss Federal Laboratories for Materials Science and Technology, Überlandstrasse 129, Dübendorf 8600, Switzerland

## Abstract

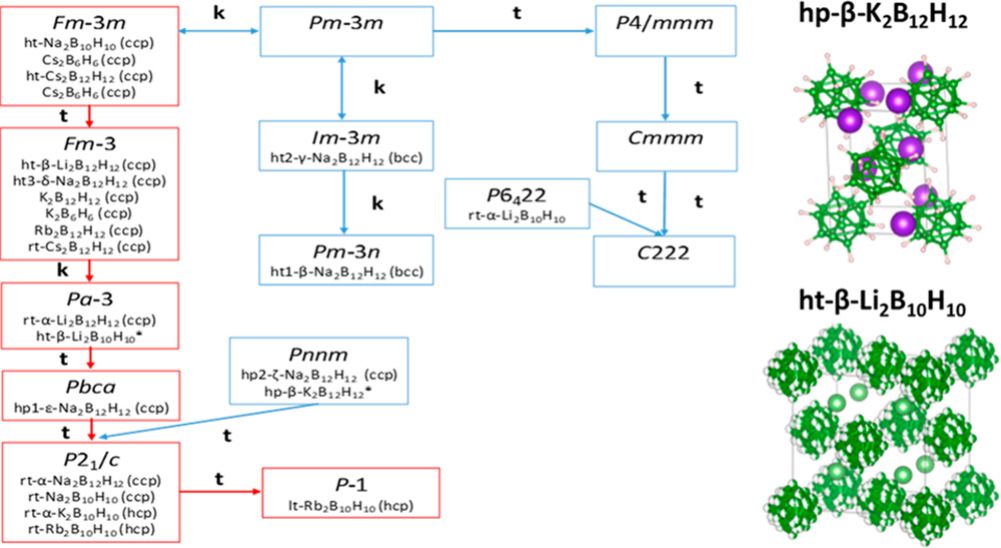

In this work, we report on the structural properties of alkali hydrido-*closo*-(car)borates, a promising class of solid-state electrolyte materials, using high-pressure and temperature-dependent X-ray diffraction experiments combined with density functional theory (DFT) calculations. The mechanical properties are determined via pressure-dependent diffraction studies and DFT calculations; the shear moduli appear to be very low for all studied compounds, revealing their high malleability (that can be beneficial for the manufacturing and stable cycling of all-solid-state batteries). The thermodiffraction experiments also reveal a high coefficient of thermal expansion for these materials. We discover a pressure-induced phase transition for K_2_B_12_H_12_ from *Fm*3̅ to *Pnnm* symmetry around 2 GPa. A temperature-induced phase transition for Li_2_B_10_H_10_ was also observed for the first time by thermodiffraction, and the crystal structure determined by combining experimental data and DFT calculations. Interestingly, all phases of the studied compounds (including newly discovered high-pressure and high-temperature phases) may be related via a group–subgroup relationship, with the notable exception of the room-temperature phase of Li_2_B_10_H_10_.

## Abstract 2

Herein, we study the pressure and temperature dependencies of alkali hydrido-*closo*-borates in extracting the mechanical properties of this class of compounds that have a promising future as solid electrolytes. In our research, we have discovered and determined two new high-pressure and high-temperature crystal structures.

## Introduction

Since the discovery of superionic conductivity in the high-temperature phase of Na_2_B_12_H_12_,^1^ there has been an increasing interest in hydrido-*closo*-(car)borates (H-*c*-B) and hydrido-*nido*-(car)borates and their solid solutions,^2−11^ as well as their halogenated derivatives.^12^ Several of these compounds or solid solutions exhibit very high ionic conductivities of >1 mS/cm at room temperature and are chemically and electrochemically very stable.^13,14^ Recently, some prototypes of all-solid-state batteries using hydrido-*closo*-(car)borates as electrolytes have demonstrated very promising performances.^15,16^ Furthermore, novel cost-effective methods have been developed to synthesize hydrido-*closo*-borates from solution,^17,18^ allowing electrode impregnation to notably improve the ionic contact between the electrode and electrolyte.^19^ These results demonstrate that this family presents many excellent properties as solid ionic conductors for new generations of all-solid-state batteries.

Understanding the bulk mechanical properties of solid electrolytes in general and H-*c*-B in particular is crucial for developing a manufacturing method for all-solid-state batteries as well as for improving their cycling stability.^20^ For example, charging and discharging cycles can induce changes in the volume of the electrode materials, which should be accommodated by the solid electrolyte without mechanically disrupting the electrode–electrolyte interface. Some of the authors have demonstrated that a solid electrolyte based on a solid solution between H-*c-*B and hydrido-*closo*-carborate is stable for at least 800 charging/discharging cycles.^21^ Mechanical properties such as the shear modulus of the solid electrolyte are also considered to be important parameters in some models of dendrite formation in solid electrolytes.^22^ Thermal expansion of the materials constituting the battery may also induce mechanical stress when the battery is subject to overheating. A colossal barocaloric effect has also been predicted for Li_2_B_12_H_12_;^23^ hence, the behaviors of this family of materials with pressure and temperature are important aspects of understanding these properties. Furthermore, while the fundamental crystal chemistry of hydrido-*closo*-borates is well established,^24^ some pieces are still missing. For instance, a temperature-induced phase transition has been observed by differential scanning calorimetry (DSC) experiments for Li_2_B_10_H_10_ and (K,Cs)_2_B_12_H_12_, though no crystal structure was given.^25,26^ In addition, only a few reports about the high-pressure behavior for this family of compounds exist, except for (Na,Cs)_2_B_12_H_12_.^27,28^ In this context, insight into their structural behavior under external stimuli (pressure and temperature) is a critical aspect for extracting some fundamental knowledge and physical properties, as the coefficients of thermal expansion (CTEs) and the isothermal compressibility, of this class of compounds. In this work, we have investigated the temperature- and pressure-dependent X-ray diffraction of a series of H-*c*-B and NaCB_11_H_12_ to provide experimental data of the thermal expansion and compressibility of constituents of solid-state sodium and lithium ionic conductors. In our investigations, we have discovered and determined two new polymorphs, namely, high-temperature (ht, for temperatures above room temperature) β-Li_2_B_10_H_10_ and high-pressure (hp) β-K_2_B_10_H_10_. These experimental results are completed by theoretical density functional theory (DFT) calculations, and a comprehensive analysis of the structural and vibrationnal behavior of these materials is given.

## Background

**Figure 1:**
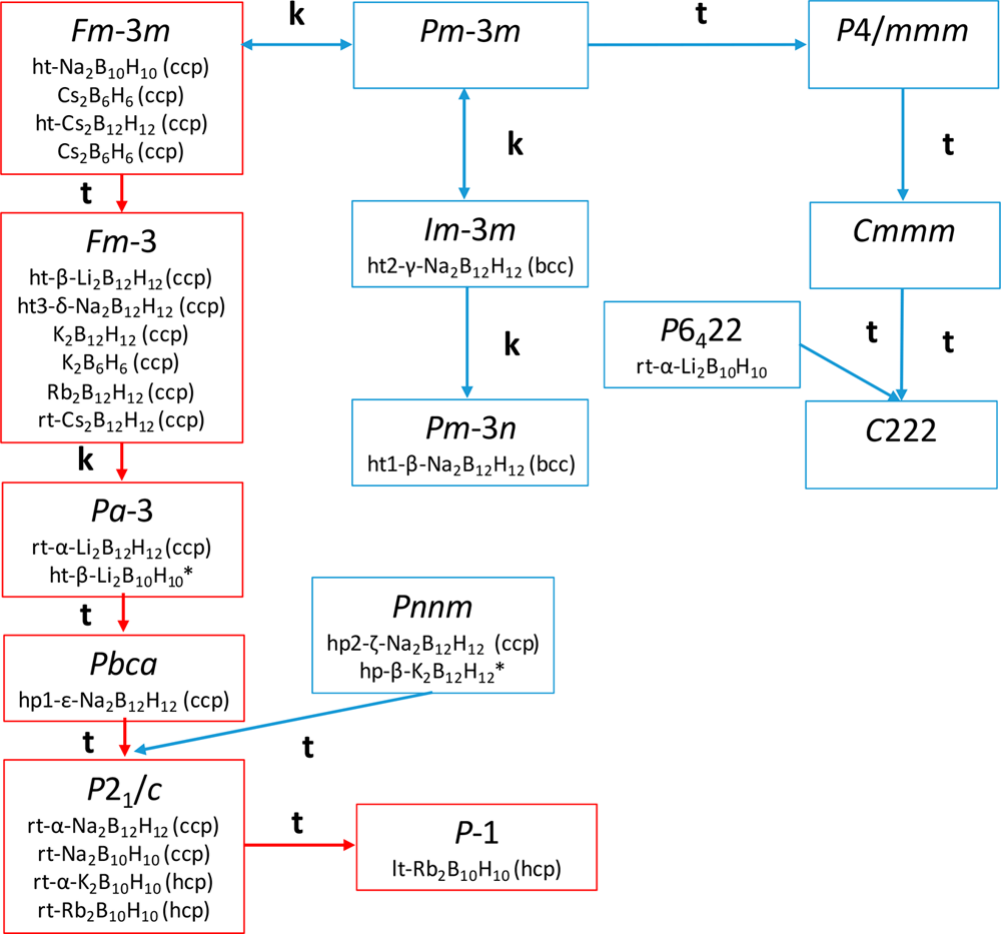
Group–subgroup relationship between the hydrido-*closo-*borates. **t** stands for the *translationengleich* subgroup, and **k** for the *klassengleich* subgroup. The asterisk indicates the crystal structures determined in this work. rt and lt stand for room temperature and low temperature (here below 250 K), respectively.

Even though H-*c*-B exhibit a very rich crystal chemistry with numerous temperature- and pressure-induced phase transitions, they share most of the time common aristotypes such as cubic close packing (*ccp*), hexagonal close packing (*hcp*), and body center cubic (*bcc*) arrangements.^24^ Along the series of alkali dodeca H-*c*-B, at ambient pressure and temperature, the larger cations (K,Rb,Cs)_2_B_12_H_12_ crystallize in the cubic *Fm*3̅ space group and Li_2_B_12_H_12_ crystallizes in the cubic *Pa*3̅ space group with undistorted *ccp* while Na_2_B_12_H_12_ adopts monoclinic *P*2_1_/*c* symmetry with the distorted *ccp*. With respect to the deca H-*c*-B, (Na,K,Rb)_2_B_10_H_10_ adopt the monoclinic *P*2_1_/*c* space group with distorted *ccp*, *hcp*, and *hcp*, respectively, whereas Li_2_B_10_H_10_ stands as an exception with hexagonal space group *P*6_4_22 without cubic or hexagonal compact underlying packing. Their temperature-induced polymorphic phase transitions have been studied and determined for (Li,Na,Rb,Cs)_2_B_12_H_12_ and Na_2_B_10_H_10_,^26,29,30^ while pressure-induced phase transitions have been investigated solely for Na_2_B_12_H_12_. It undergoes two-phase transitions at relatively low pressures: *P*2_1_/*c* → [0.3–0.8 GPa] *Pbca* → [5.7–8.1 GPa] *Pnnm*.^27^Figure 1 shows the different symmetries encountered for all of the known H-*c*-B together with their underlying packing in a group–subgroup graph. It is worth noting that most of the H-*c*-B exhibit a direct group–subgroup relationship (red path in Figure 1) that can come into play for the phase transitions for these compounds as discussed in detail for the pressure-induced transitions of Na_2_B_12_H_12_.^27^ Furthermore, the preferred packing for this family is *ccp* with the exception of ht polymorphs of Na_2_B_12_H_12_ adopting the *bcc* packing for ht1-β-Na_2_B_12_H_12_ and ht2-γ-Na_2_B_12_H_12_. Na_2_B_12_H_12_ possesses the richest phase diagram among all of the H-*c*-B, and it is the only one found in the *bcc* arrangement that is known to favor ionic conductivity.^31^

## Results and Discussion

### Pressure Dependence

#### High-Pressure X-ray Diffraction

**Figure 2:**
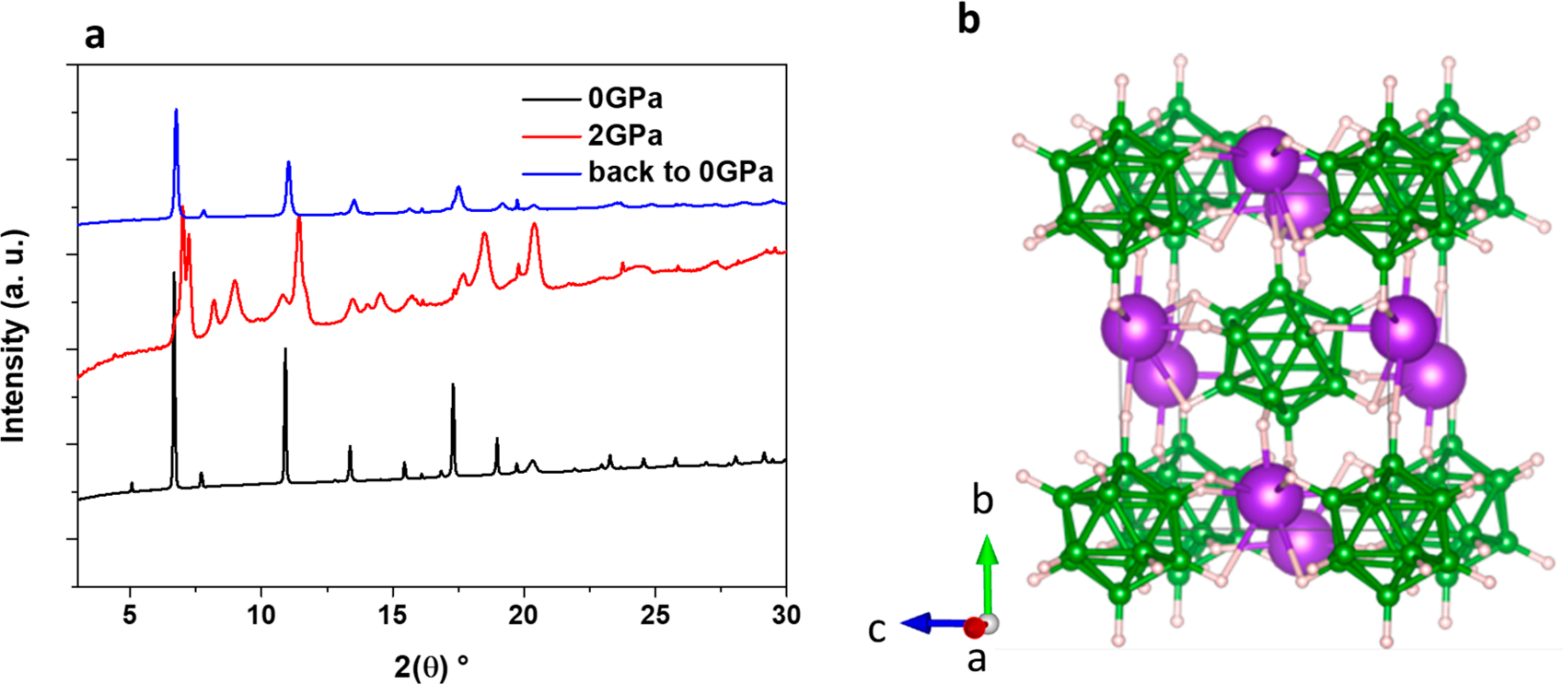
(a) Diffraction patterns of K_2_B_12_H_12_ at 0 GPa (black), 2 GPa (red), and back to ambient pressure (blue) depicting the reversible pressure-induced phase transition. (b) Representation of the hp-β-K_2_B_12_H_12_ crystal structure.

Six different samples [(Li,Na,K)_2_B_12_H_12_, (Li,K)_2_B_10_H_10_, and NaCB_11_H_12_] were investigated at Swiss Norwegian Beamline (SNBL) to study their behavior under pressure. Except for Na_2_B_12_H_12_, for which the hp phase transitions were already described,^27^ K_2_B_12_H_12_ also undergoes a reversible phase transition at >2 GPa toward a polymorph isostructural to hp2-ζ-Na_2_B_12_H_12_ with orthorhombic *Pnnm* symmetry (Figure 2a). The cell parameters were first determined using Pawley refinement with hp2-ζ-Na_2_B_12_H_12_ as first input and manually increased to fit the diffraction pattern; once a good approximation was found, the refinement was carried out. The Rietveld refinement was subsequently achieved with the as-obtained cell parameters and hp2-ζ-Na_2_B_12_H_12_ atomic positions that enabled us to obtain hp-β-K_2_B_12_H_12_ with the following cell parameters: *a* = 7.1670(13) Å, *b* = 9.212(6) Å, and *c* = 7.560(3) Å (Figure 2b). Despite the low quality of the pattern, due to the strains and preferential orientations induced by the pressure, refinement successfully converged with the following reliability factors: *R*_wp_ = 1.88, *R*_p_ = 1.16, and goodness of fit (GoF) = 6.5 (Figure S1).

Owing to the low quality of the diffraction pattern, DFT calculations were performed to further confirm the stability of the *Pnnm* symmetry. The diffraction peak at 8.2° cannot be explained by the refinement, which can be due to the remaining 111 reflection from the lp phase or another polymorph. The calculated pressure dependence of the free energy (*F* = *E*_0_ + *pV*) reveals the phase transition of K_2_B_12_H_12_ (*Fm*3̅ → *Pnnm*) at 3.58 GPa, hence further confirming the experimental data (Figure S2). The phase transition is accompanied by an ∼7% specific volume change indicating a first-order transition. While a group–subgroup relationship exists between *Fm*3̅ and *Pnnm*, a direct comparison of both structures does not allow identification of the transition mechanism. A transformation of the hp-β-K_2_B_12_H_12_*Pnnm* phase into *P*2_1_/*c*, with *P*2_1_/*c* ⊂ *Fm*3̅, using matrices **a**_**mono**_ = −**b**_**ortho**_ – **c**_**ortho**_, **b**_**mono**_ = **a**_**ortho**_, and **b**_**mono**_ = −**b**_**ortho**_ + **c**_**ortho**_ with an origin shift **c**_**mono**_ = **c**_**ortho**_ + ^1^/_2_ (Figure S3) was performed prior to the comparison. The phase transition is diplacive combining a diffusionless (martensitic-like) transformation for the B_12_H_12_^2–^ units with the displacement of the potassium cation like Na_2_B_12_H_12_.^27^ The martensitic-like transition is displayed in Figure S4, during which the cubic lattice is transformed into the monoclinic one. The deformation leads to the following high values of the Lagrangian strain tensor with *e*_11_ = *e*_33_ = 0.1515, *e*_22_ = −0.2643, and *e*_31_ = *e*_13_ = 0.1272, which must be taken into account to treat the phase transition using a finite strain approach. As a consequence, the Landau free energy must be built with an order parameter–strain coupling.^32^ Using group theory analysis with amplimode,^33,34^ on the Bilbao Crystallographic Server, one can identify that the decrease in symmetry from *Fm*3̅ to *P*2_1_/*c* is driven by three one-dimensional irreducible representations (irreps) Γ^4+^ at wave vector *k* (0, 0, 0), X^+^, and X^2+^ and one three-dimensional X^2+^ irrep at *k* (0, 1, 0).

#### Mechanical Properties

In the pressure range of 0–6 GPa, Li_2_B_12_H_12_, (Li,K)_2_B_10_H_10_, and NaCB_11_H_12_ do not undergo a pressure-induced phase transition. Together with those of hp2-ε-Na_2_B_12_H_12_ and hp-β-K_2_B_12_H_12_, their cell volumes were determined as a function of pressure. When the data allowed, experimental bulk moduli were determined by fitting the Murnaghan equation of state (eq 1) with the experimental data (Figure S5), and the results are listed in Table 1.
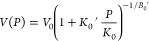
1

**Table 1:** Summary of the Bulk (*K*) and Shear (*G*) Moduli Obtained Experimentally and by DFT of the Alkali Deca and Dodeca H-*c*-B^a^

compound	space group	*G*_r_ (GPa)	*G*_v_ (GPa)	*K*_r_ (GPa)	*K*_v_ (GPa)	*K*_exp_
Li_2_B_10_H_10_	*P*6_4_22 (No. 181)	6.20	7.43	20.01	20.46	16.3(4.6)
Li_2_B_12_H_12_	*Pa*3̅ (No. 205)	11.31	8.96	20.84	20.84	21.3(1.4)
Na_2_B_10_H_10_	*P*2_1_/*c* (No. 14)	8.35	7.21	19.08	19.24	–
Na_2_B_12_H_12_	*P*2_1_/*c* (No. 14)	2.11	6.08	7.20	16.10	13.1(6)
K_2_B_10_H_10_	*P*2_1_/*c* (No. 14)	8.38	7.24	19.07	19.21	25.5(2.5)
K_2_B_12_H_12_	*Fm*3̅ (No. 202)	7.41	5.99	16.85	16.49	–
Rb_2_B_10_H_10_	*P*2_1_/*c* (No. 14)	5.25	4.45	17.10	17.56	–
Rb_2_B_12_H_12_	*Fm*3̅ (No. 202)	7.34	5.94	16.80	16.80	–
Cs_2_B_10_H_10_	*P*2_1_/*c* (No. 14)	2.33	3.14	10.25	15.27	–
Cs_2_B_12_H_12_	*Fm*3̅ (No. 202)	6.17	4.91	14.68	14.68	–

a The indices r and v stand for the Reuss and Voigt limits, respectively.

The experimental bulk moduli determined for the compounds mentioned above are in the range of 16.3–25.5 GPa, revealing very high compressibility in good agreement with our previous study of Na_2_B_12_H_12_.^27^

**Figure 3:**
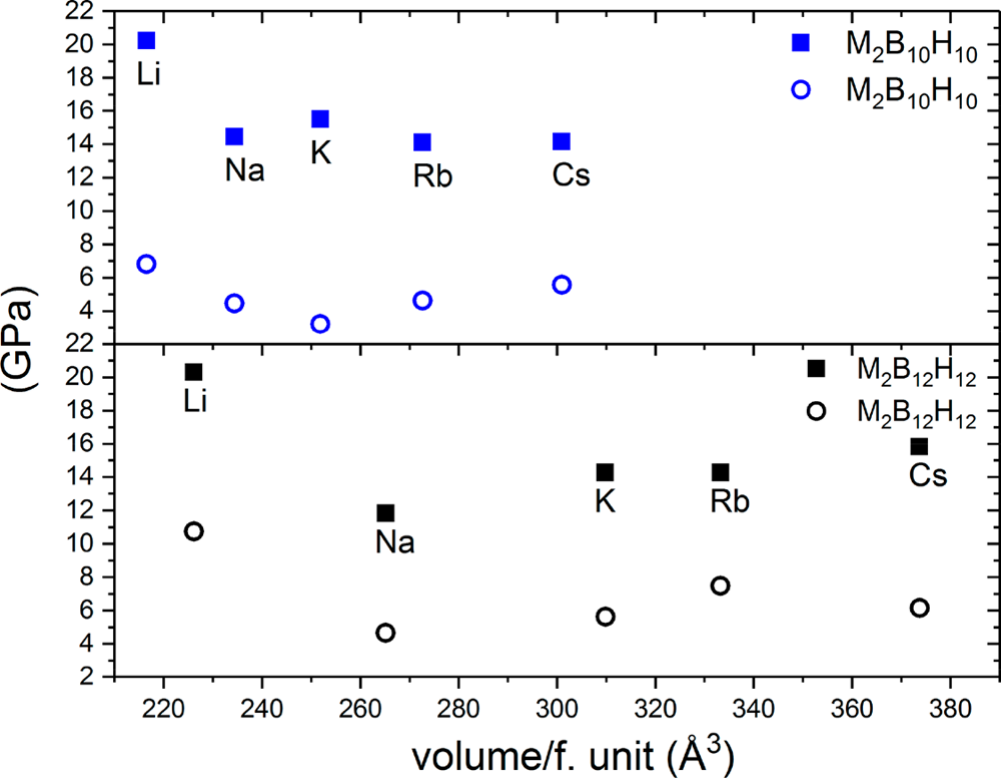
Calculated bulk (squares) and shear (circles) moduli for alkali metal deca and dodeca H-*c*-B. The average (*X*_r_ + *X*_v_)/2 values are presented.

The elastic properties of the ordered phases of alkali H-*c*-B were also determined by DFT calculations (Table 1). They are in good agreement with experimental values, validating our calculation strategy. One has to keep in mind that calculated values correspond to the adiabatic constants while experimental data are for isothermal values. The evolution of the bulk (*K*) and shear (*G*) moduli as a function of the volume per formula unit is represented in Figure 3. For the Li cation, the bulk and shear moduli are systematically larger than for other elements of the group. Along the series M_2_B_10_H_10_, the bulk modulus somewhat decreases for heavier cations, while for the M_2_B_12_H_12_ family, one can observe a slight increase with the mass of the cation. The shear modulus does not follow any obvious trend; however, it is significantly smaller than for oxides or sulfides (>10 GPa).^35^ This is an indication of the malleability of these compounds, especially with dodeca H-*c*-B anions; for example, following the Pugh criterion for ductile materials affords a *K*/*G* ratio of >1.75.^36^

The relatively large compressibility and malleability of H-*c*-B are beneficial for solid-state battery manufacturing, making it easier to densify the solid electrolyte layer and to achieve intimate contact with the electrode. If a good contact between the H-*c*-B and active material is established (via solution processing, for example),^19^ it can be maintained upon cycling because of their high deformability. It is worth mentioning that mechanical properties obtained from structural studies cannot always be translated to bulk properties in a battery where a solid electrolyte is typically a pressed polycrystalline powder. Nevertheless, H-*c*-B have proven to maintain stable interfaces in all-solid-state batteries after many cycles, including without the application of significant external mechanical pressure.^21^ The experimental and theoretical values appear to be characteristic for the entire series of compounds and can thus be extrapolated to predict the behavior of new ionic conductors based on H-*c*-B and derived compounds. Additionally, the very low shear modulus is an indication that such materials easily adapt to the structural changes of the electrode.

### Temperature Dependence

#### High-Temperature X-ray Diffraction

**Figure 4:**
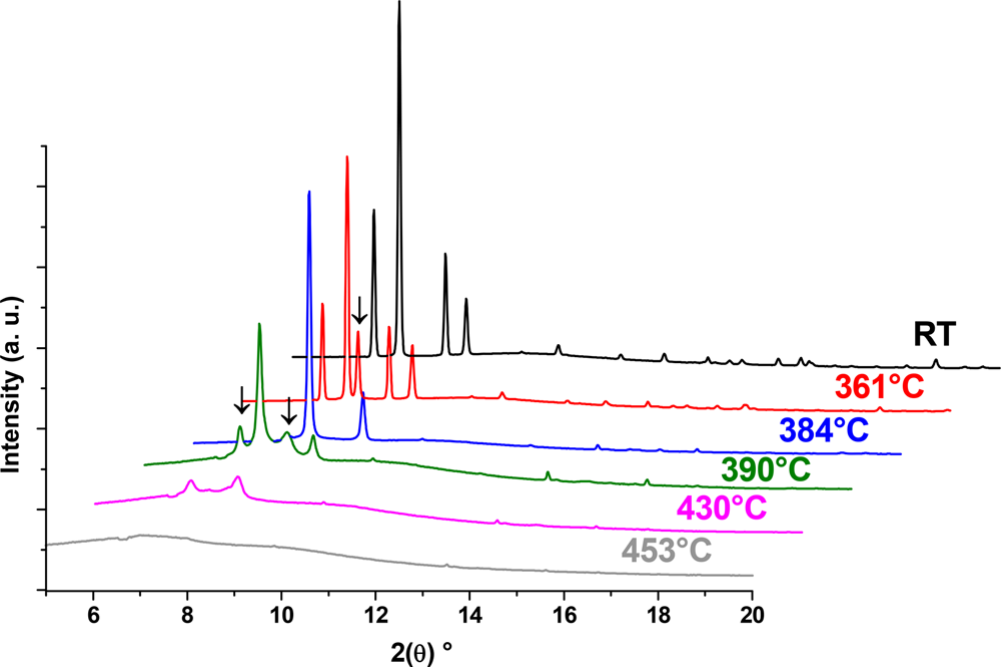
Temperature dependence of the diffraction patterns for Li_2_B_10_H_10_. At room temperature, only rt-α-LiB_10_H_10_ is present. At 361 °C, ht-β-LiB_10_H_10_ appears (arrow), and both polymorphs coexist up to 384 °C. At 390 °C, the unidentified phase appears (arrow), and both phases coexist up to 430 °C. At 453 °C, amorphization of the material occurs.

Most of the crystal structures of deca and dodeca H-*c*-B of alkali metals are known for their low- and high-temperature polymorphs,^24,26,30^ with the exceptions being the high-temperature phases of Li_2_B_10_H_10_ and (K,Rb)_2_B_12_H_12_, which were observed by DSC measurements, but the structure has never been determined.^25,26^ K_2_B_12_H_12_ undergoes a phase transition at ∼540 °C, which is around the transformation temperature of the glass capillary; hence, the transition was not recorded during our experiment. Nonetheless, we did observe a phase transition for rt-α-Li_2_B_10_H_10_ starting to transform into ht-β-Li_2_B_10_H_10_ at 361 °C. From this temperature, both polymorphs coexist up to 384 °C, at which rt-α-LiB_10_H_10_ totally transforms into ht-β-LiB_10_H_10_. At 390 °C, the diffraction peaks of ht-β-LiB_10_H_10_ start to decrease with the appearance of an amorphous and a new, crystal phase. The possible nature of this new crystal structure will be discussed below. From 430 °C, only the new and amorphous phases are present up to 453 °C, the temperature at which the compound becomes amorphous. Figure 4 displays the diffraction patterns for the different steps described. These observations are in good agreement with the previous study of the thermal behavior of Li_2_B_10_H_10_, in which an entropically driven order–disorder phase transition was suggested.^25^ However, the appearance of the unidentified crystal phase was never reported; due to the low quality of the diffraction pattern, a direct structural determination was not possible during our experiments, but the structure was likely determined with the support of DFT calculations (see below).

With regard to ht-β-Li_2_B_10_H_10_, the phase appears to be isostructural to Li_2_B_12_H_12_, and its pattern can be indexed with a cubic lattice with *a* = 9.5316(3) Å and *V* = 865.96(7) Å^3^. The structure can be refined in two different space groups, *Fm*3̅*m* and *Pa*3̅, with similar agreement factors (*R*_wp_ = 4.2 and 4.0 for *Pa*3̅ and *Fm*3̅*m*, respectively). In both structures, the B_10_H_10_^2–^ ions are orientationally disordered as suggested in the previous study.^25^

#### DFT Calculations for ht-β-Li_2_B_10_H_10_

**Figure 5:**
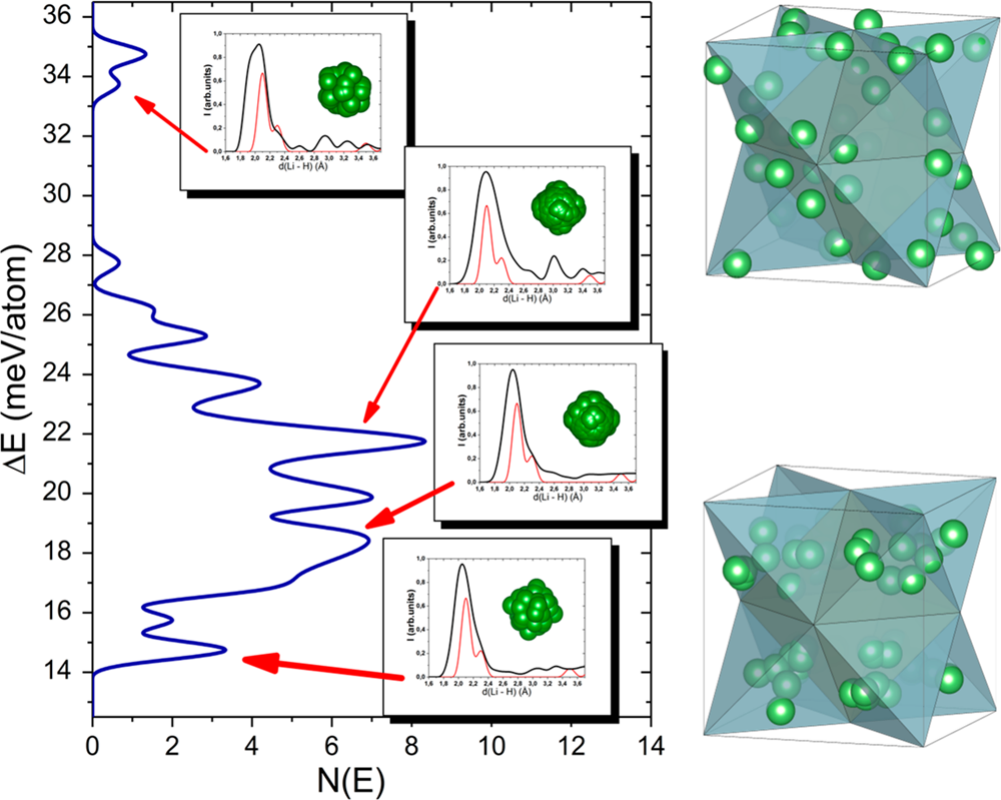
Energy distribution of Li_2_H_10_H_10_ in the cubic *ccp* structure with respect to the *hcp* (*P*6_4_22) ground state. The insets show radial distribution functions for the Li–H separation; the red line is for the reference hexagonal structure. The green spheres are projections of anions on the (*a, b*) plane for the given energy range. The right side shows the distribution of cations corresponding to the configurations with the lowest and highest energies; for the sake of simplicity, only coordination tetrahedra of the *fcc* lattice are colored light blue.

These structures differ in the average orientation of B_10_H_10_^2–^ anions, as shown in Figure S6. For the structure with *Pa*3̅ symmetry, the B_10_H_10_^2–^ anions are oriented such that the longer anion axis is along one of the principal lattice directions (three preferred orientations). This results in an average quasi-octahedral shape. For *Fm*3̅*m* symmetry, there are four orientations along the cubic unit cell diagonals preferred by B_10_H_10_^2–^ anions. They average to the effective cubic shape of the anion (see Figure S6). Because both geometrical figures, the cube and the octahedron, have the same number of symmetry elements, the distinction between the two crystal structures of Li_2_B_10_H_10_ must be related to the positions of cations, which is coupled to the anion orientation. To determine which of the two orientations are preferred (higher cohesive energy), we performed series of DFT calculations. Because the high-temperature phase is disordered, the procedure for the calculations was developed. The structures with random anion orientations/cation distribution were used to calculate the energy; the details are presented in the Supporting Information. The energy distribution for the atomic configurations in the cubic phase of Li_2_B_10_H_10_ is presented in Figure 5. It consists of separated energy maxima starting with a Δ*E* of 0.014 eV/atom above the *P*6_4_22 ground-state energy up to a Δ*E* of 0.035 eV/atom for the least stable configurations. For four selected regions, the radial distribution functions (rdfs) for Li–H separation were calculated, as shown in the insets of Figure 5. In general, the rdf for the most stable configurations resembles that of the low-temperature phase, where two Li–H distances of 2.1 and 2.3 Å are present (they are larger than the values of 2.028, 2.044, and 2.216 Å reported for the experimental structure of Li_2_B_10_D_10_).^25^ For the least stable configurations, the Li–H separation strongly differs from the low-temperature one. The Li–H spacing has a broad distribution within the range of 1.9–2.7 Å. Such short interatomic distances indicate that Li is closely connected to anions. This can be seen in Figure 5 where the distribution of cations is shown for the structure with the lowest energy, and cations were confined to the tetrahedral interstitial voids. The cations with the least stable configuration are located at tetrahedral facets rather than in the tetrahedral center. The projection of B_10_H_10_^2–^ anion orientations on the (*a, b*) crystal plane indicates they are oriented with a longer axis along the (100), (010), or (001) lattice direction except for the least stable structure, where the orientation is along the unit cell diagonals (see Figure 5). This points toward *Pa*3̅ space group symmetry for the high-temperature phase of Li_2_B_10_H_10_ and a possible second phase transition to the *Fm*3̅*m* space group at higher temperatures prior to the thermal decomposition of the compound. Having this in mind, one can suggest that the unidentified ht phase could be the cubic *Fm*3̅*m* phase. A Rietveld refinement was then performed with an *a* of 10.219(3) Å; however, owing to the poor quality of the diffraction pattern, the fit was not optimal, but the solution cannot be excluded. Additional work would be necessary to demonstrate this last transition.

#### Coefficient of Thermal Expansion

CTEs were determined for several alkali hydrido-*closo*-(car)borates studied here by fitting the evolution of the volume as a function of the temperature to a polynomial function (eq 2). The CTE α can be determined using eq 3. The results are listed in Table 2.
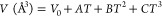
2
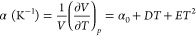
3

**Table 2:** Summary of the Coefficients (*V*_0_, *A*, *B*, and *C*) of the Polynomials Used to Fit the Evolution of the Volume of the Cells as a Function of Temperature Together with the Coefficients (α_0_, *D*, and *E*) of the Equations of the CTE as a Function of Temperature and the Averages of the CTE [α(avg)] Calculated for the Indicated Temperature Range

compound	space group	*V*_0_ (Å^3^)	*A*	*B*	*C*	α(avg) (K^–1^)	α_0_ (K^–1^)	*D* (K^–2^)	*E* (K^–3^)	*T* (K)
Li_2_B_10_H_10_	*P*6_4_22	600.9(3)	0.020(1)	3.9(1) × 10^–5^	–	0.95 × 10^–4^	3.333(3) × 10^–5^	1.342(1) × 10^–7^	–1.67(1) × 10^–11^	320–620
Na_2_B_10_H_10_	*P*2_1_/*c*	870.8(3)	0.123(1)	–	–	1.35 × 10^–4^	1.410(1) × 10^–4^	–	–	300–380
Na_2_B_10_H_10_	*Fm*3̅*m*	891.2(4)	0.1376(7)	–	–	1.4 × 10^–4^	1.533(7) × 10^–4^	–	–	400–795
Na_4_(B_10_H_10_)(B_12_H_12_)	*Fm*3̅*m*	900.5(6)	0.468(3)	–5.50(7) × 10^–4^	3.19(4) × 10^–7^	1.8 × 10^–4^	4.69(1) × 10^–4^	–1.098(3) × 10^–6^	9.32(2) × 10^–10^	300–796
K_2_B_12_H_12_	*Fm*3̅	1141(1)	0.1838(2)	–	–	1.4 × 10^–4^	1.90(3) × 10^–5^	–	–	305–724
NaCB_9_H_10_	*Pna*2_1_	406.1(2)	0.1129(4)	–	–	2.5 × 10^–4^	2.751(1) × 10^–4^	–	–	325–500
Na_2_B_12_H_12_	*P*2_1_/*c*	492	0.19	–3.8 × 10^–4^	3.67 × 10^–7^	1.3 × 10^–4^	3.902 × 10^–4^	7.723 × 10^–7^	7.459 × 10^–10^	273–540
Na_2_B_12_H_12_	*Pm*3̅*n*	457.9	0.12	–	–	2.3 × 10^–4^	2.621 × 10^–4^	–	–	540–580
Na_2_B_12_H_12_	*Im*3̅*m*	471.4	0.24	–	–	1.8 × 10^–4^	5.091 × 10^–4^	–	–	580–700
Na_2_B_12_H_12_	*Fm*3̅*m*	1051.1	0.14	–1.88 × 10^–5^	–	1.1 × 10^–4^	1.109(1) × 10^–4^	–1.075(6) × 10^–11^	–	540–750

The relatively high value of the CTE, compared to those of oxides that are usually 2 orders of magnitude lower than the values of this family of compounds, points out a strong dilatation for these materials with temperature. This feature could induce mechanical stresses between the different components of the battery cathode (usually oxide-based), anode, and electrolyte. These mechanical stresses would be detrimental in the case of overheating. However, the very low shear moduli for these compounds could overcome this issue; the material will flow and with proper construction hence shall not affect the interface. Low shear moduli can be seen as the behavior of the materials, with external stimuli, approaching that of the liquid. Additionally, elastic constants (Table 1) indicate the malleability of this class of materials. The thermal expansion is a manifestation of the anharmonicity of the lattice vibrations, and fitting the evolution of the volume as a function of the temperature with a polynomial on the order of ≥2 indicates a strong anharmonicity, related to the orientational disorder of anions.

#### Structural and Vibrational Analysis

Symmetry analysis reveals relations between space group symmetry of all alkali metal deca and dodeca H-*c*-B. The low-temperature hexagonal phase of Li_2_B_10_H_10_ is somehow an exception from the family of structures originating from the *Pm*3̅*m* space group.

**Figure 6:**
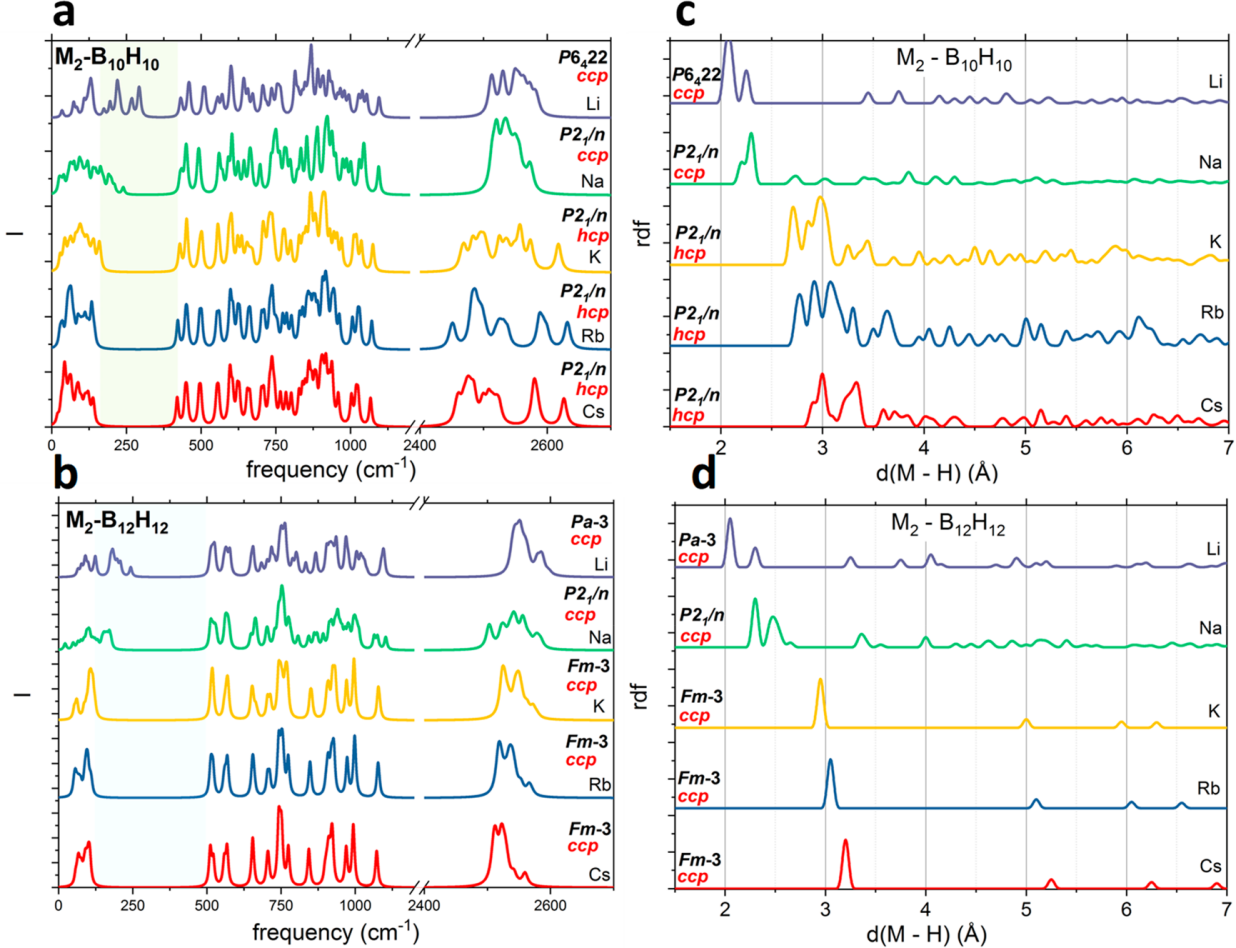
Distribution of the phonons at the Γ point for alkali metal (a) deca H-*c*-B and (b) dodeca H-*c*-B and the radial distribution function for cation–hydrogen separation for alkali metal (c) deca H-*c*-B and (d) dodeca H-*c*-B. The calculated frequencies are broadened with a 5 cm^–1^ Lorentzian.

**Figure 7:**
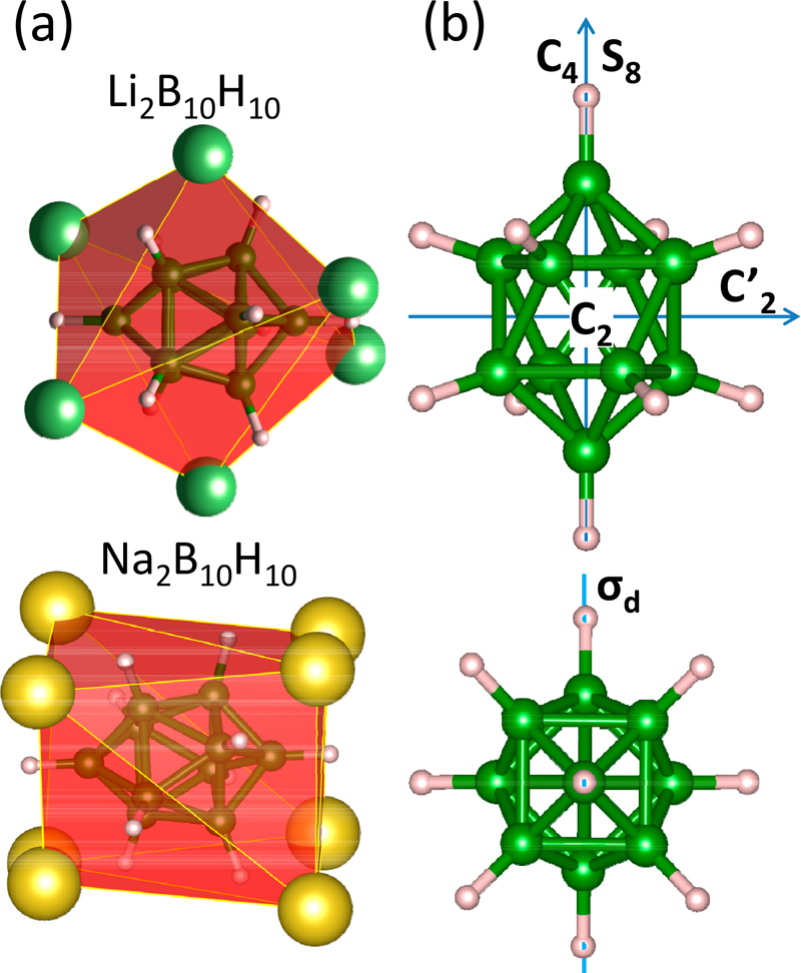
(a) Coordination polyhedra for B_10_H_10_^2–^ anions in alkali metal deca H-*c*-B. Small green and gray spheres represent boron and hydrogen, respectively; large green spheres represent Li, and large yellow spheres Na. (b) Schematic view of the symmetry elements of the *D*4*d* point group of the B_10_H_10_^2–^ anion. C_4_ and S_8_ are rotations/improper rotations around the 4- and 8-fold axes, respectively. C_2_ and C′_2_ are rotations around the 2-fold axis, and σ_d_ stands for the mirror plane.

To improve our understanding of the similarities among members of this family of compounds, below we report on structural and vibrational analysis. In Figure 6, the distribution of the brillouin zone center modes for alkali metal deca and dodeca H-*c*-B together with the radial distribution function for metal–hydrogen separation is displayed. With regard to deca H-*c*-B, a clear division into two groups is apparent. Li and Na show a short separation between hydrogen and metal that can be correlated with the broader extent of the lattice modes. Especially for lithium, the lattice modes go beyond 250 cm^–1^, which is an indication of direct and strong Li–B_10_H_10_^2–^ interaction. More detailed information about internal B_10_H_10_^2–^ vibrations can be found elsewhere;^37,38^ however, the splitting of B–H modes (>2400 cm^–1^) is related to the bond distortion, and the internal *closo*–cage vibrations in the range of 400–1200 cm^–1^ are modified by small deformations of the anion. The largest splitting of the highest-frequency modes (B–H stretching modes) is observed for the heaviest cations, K, Rb, and Cs. This is related to the *hcp* packing of anions,^24^ rather than the *ccp* packing that is observed for Li and Na, and the symmetry of the B_10_H_10_^2–^ molecule. This anion has point group symmetry *D*4*d* as for the capped square antiprism (see Figure 7). The distribution of cations is compatible with C_4_ and S_8_ symmetry elements of the molecule; thus, the differences can be ascribed to the packing of anions.

For these modes, similarities for K, Rb, and Cs are visible, which would be expected as these compounds have the same symmetry for the same anions. A large splitting of B–H modes for K, Rb, and Cs deca H-*c*-B should be noticed; they are related to the distribution of these cations in the lattice. For compounds with Li and Na, B–H stretches have distinct splittings of frequencies.^38^ While the spectra related to the internal vibrations of anions have similarities within each class of compounds, the differences are related to the different site symmetry of the anion in Li- and Na-containing compounds. The largest differences between them are present in the upper range of lattice modes above 150 cm^–1^. While for K, Rb, and Cs a clear gap between the lattice and internal anion modes is present, this gap is smaller for Li and Na, especially for Li_2_B_10_H_10_, where lattice vibrations are present above 250 cm^–1^; for Na, they are less extended, and for the icosahedral dodeca anion, they extend to lower frequencies.

In panels c and d of Figure 6, the pair distribution function calculated for metal–hydrogen separation is presented. For both classes of compounds, clear differences are visible between Li/Na and heavier metals. While a well-defined Li–H separation of just >2 Å is visible for Li_2_B_10_H_10_, these separations are still present in Na_2_B_10_H_10_ with some additional peaks below 3 Å. All heavier alkali metals are separated from the nearest hydrogen by >2.5 Å, and the distribution of M–H distances is not well-defined for deca H-*c*-B (Figure 6c), showing a bond length distribution between 2.5 and 6 Å. The opposite is observed in dodeca H-*c*-B as the M–H spacing is well-defined for Cs, Rb, and K and decreases with the decrease in mass (radius) of the metal cation (Figure 6d). For Li and Na in dodeca H-*c-*B, this spacing is smaller (∼2 Å) and the distance distribution similar to that in deca H-*c*-B is clear, also for sodium.

The short metal–hydrogen distances for the two lightest metals are correlated with a broader range of their lattice modes and indicate direct M–H interaction. This is most apparent for Li_2_B_10_H_10_. From the Pauling rules for ionic compounds, the coordination of metals can be estimated from the ratio of ionic radii of anions and cations.^39^ This is particularly well observed in metal hydridoborates, where one can assume B_10_H_10_^2–^ radii of 6.0 Å (5.8 Å for B_12_H_12_^2–^). The size of the alkali metal cations increases with atomic number and according to Shannon radii is^40^ 1.2 Å for Li, 1.9 Å for Na, 2.66 Å for K, 2.96 Å for Rb, and 3.38 Å for Cs. The ionic size ratio for compounds with B_10_H_10_^2–^ anions is 0.20 for Li (3), 0.32 for Na (4), 0.44 for K (6), 0.49 for Rb (6), and 0.56 for Cs (6); numbers in parentheses indicate coordination numbers for anions. For compounds with B_12_H_12_^2–^ anions, the formal coordination numbers are the same. In fact for all of these compounds, the cations are located within coordination tetrahedra between the nearest anions, as even for heavier alkali metals the ratio is close to 0.414, which is the limit of tetrahedral coordination. The structure analysis indicates that in the *P*2_1_/*c* structure of (K,Rb,Cs)_2_B_10_H_10_ half of the cations are located at octahedral voids. The exceptions are Li and Na, where each cation is surrounded by three anions and thus is located at the face of coordination tetrahedra. The relation between the coordination number and lattice type is known to correlate with ionic conductivity,^31,41^ and *bcc* anion packing is the ultimate for the best ion conductor.

In Figure 7, we present coordination polyhedra for B_10_H_10_ anions with Li and Na. Such a presentation reveals highly symmetric polyhedra for Li and Na. The positions of cations follow the *D*4*d* symmetry of the anion, forming a deformed cubic coordination for Na_2_B_10_H_10_, and six Li cations surround the anion in Li_2_B_10_H_10_. Among the symmetry elements of the *D*4*d* point group of the capped square antiprism that is B_10_H_10_, only those related to rotations are accessible due to thermal excitations (see Figure 7). Improper rotation by 45° (S_8_) without reflection is the less energy demanding process that preserves the orientation of anions in the crystal opening additional sites for cations. This process will not change the hexagonal symmetry of the low-temperature phase. Rotation around one of the C_2_ axes by 90° changes the orientation of the anion in the crystal lattice and thus breaks the *a,b,a,b* stacking of the *hcp* lattice. This is in fact observed in the high-temperature *ccp* structure of this compound, where the anions are still aligned along principal lattice directions with cations distributed in the tetrahedral void with similar Li–H separations as in the LT phase. The strong Li–H interaction in Li_2_B_10_H_10_ is also related to low thermal expansion of this compound within the H-*c*-B class (see Table 2). As shown in Figure 6c for any configuration considered in the cubic phase, the shortest distance between hydrogen and lithium does not increase above 2.1 Å, which is consistent with the fact that with an increasing separation between anions the compounds disintegrate into molecular entities consisting of cations and anions.

The phase transition of Na_2_B_10_H_10_ from the low-temperature *P*2_1_/*c* structure to the cubic *Fm*3̅*m* one is related to the orientational disorder of anions, while eight Na^+^ ions effectively form regular cubic coordination around an anion (Figure 7). This is related to disorder in the cationic sublattice but not to changes in the anion coordination number.

## Conclusion

In this work, a class of compounds, hydrido-*closo*-borates, has been structurally investigated using *in situ* X-ray diffraction methods under external pressure and temperature stimuli, combined with DFT calculations. Those materials have demonstrated very high compressibility and very low shear moduli, revealing highly malleable materials that would allow fast structural reconstruction under mechanical stresses. Furthermore, this family of compounds has exhibited very high CTEs, 2 orders of magnitude higher than those of oxides. Interestingly, our investigations reveal two new crystal phases, the first one for K_2_B_12_H_12_ resulting from the pressure-induced phase transition around 2 GPa toward *Pnnm* symmetry and the second phase for which the transition is induced by temperature and transforms Li_2_B_10_H_10_ from *P*6_4_22 to *Pa*3̅ symmetry as suggested by DFT calculations. This study allows us to acquire a complete understanding of the crystal chemistry of this astonishing class of compounds and further confirms their trend for the *ccp* underlying anion packing.

## Experimental Section

### Powder X-ray Diffraction

Samples were purchased from Katchem Co. The sample was measured at Swiss-Norwegian Beamline BM01 of the European Synchrotron Radiation Facility in Grenoble, France. A two-dimensional (2D) image plate detector (Pilatus 2M) positioned 411 mm from the sample was used with a wavelength of 0.71414 Å. The 2D diffraction patterns were integrated with Bubble software.^42^ The sample detector geometry was calibrated with a LaB_6_ NIST standard. For high-pressure experiments, the Diamond Anvil Cell (DAC), with a flat culet with a diameter of 600 μm, was loaded in an argon-filled glovebox (MBraun, <0.1 ppm O_2_, <0.1 ppm H_2_O). The samples were loaded with ruby crystals, for pressure calibration, into a 250 μm hole drilled in a stainless-steel gasket. No pressure-transmitting medium was used because of the low bulk modulus of these families of materials. For high-temperature experiments, the samples were loaded in a 0.5 mm glass capillary in the glovebox. The temperature was controlled using a Cyberstar hot blower. For hp-β-K_2_B_12_H_12_ and ht-β-Li_2_B_10_H_10_, the structures were determined using the isostructural models of hp3-ζ-Na_2_B_12_H_12_ and rt-α-Li_2_B_12_H_12_. The cell parameters were manually adjusted prior to their refinement using the Pawley algorithm implemented in TOPAS;^43^ this algorithm was used also for the refinement of the cell parameters as a function of temperature and pressure. The refinements of the structure were performed using the Rietveld method,^44^ in TOPAS.^43^ For the hp polymorph, a spherical harmonic approach was used to simulate the strong preferential orientation caused by the DAC. The cell parameters as a function of temperature and pressure were refined using the Pawley algorithm.

### DFT Calculations

Calculations were performed within DFT with a periodic plane wave basis set as implemented in Vienna ab initio Simulation Package.^45,46^ The following calculation parameters were used: cutoff energy for basis set expansion of 700 eV, k-point sampling density (*ka*) of ≥20, convergence criterion for the electronic degrees of freedom of 10^–6^ eV/A, and for the structural relaxations the conjugated gradient method with a convergence of 10^–2^ eV/A. Projector-augmented wave potentials (PAW)^47,48^ were used for atoms with electronic configurations of 1s^1^ for H, 2s^2^2p^1^ for B, 1s^2^2s^1^ for Li, 2p^6^3s^1^ for Na, 3p^6^4s^1^ for K, 4p^6^5s^1^ for Rb, and 5p^6^6s^1^ for Cs. The gradient-corrected (GGA) exchange-correlation functional and the nonlocal corrections accounting for a weak dispersive interactions were applied.^49−51^ The normal modes at the Γ point were calculated in real space with atomic displacements of ±0.1 Å in all symmetry inequivalent directions and visualized by placing Lorentzians with a half-width of 5 cm^–1^ for each mode. The normal mode frequencies were obtained by direct diagonalization of the dynamical matrix obtained from the forces calculated for displaced configurations. Elastic constants were calculated via deformation of the unit cell, ±1% in each relevant direction and angle. For normal mode and elastic properties, fully optimized structures were used.

## Supporting Information Available

Calculation procedure for ht-Li_2_B_10_H_10_, the result of the Rietveld refinement, the free energy as a function of the pressure and the transformation of the structures from *Pnnm* to *P*2_1_/*c* for hp-K_2_B_12_H_12_ (Figures S1–S4), evolution of the cell volume as a function of pressure (Figure S5), and orientation of B_10_H_10_^2–^ anions of ht-Li_2_B_10_H_10_ with *Fm*3̅*m* and *Pa*3̅ space groups (Figure S6) (PDF)

## Accession Codes

CCDC 2123207 and 2123209 contain the supplementary crystallographic data for this paper. These data can be obtained free of charge via www.ccdc.cam.ac.uk/data_request/cif, or by emailing data_request@ccdc.cam.ac.uk, or by contacting The Cambridge Crystallographic Data Centre, 12 Union Road, Cambridge CB2 1EZ, UK; fax: +44 1223 336033.
