# Supplementary material for: Study of the Temperature- and Pressure-Dependent Structural Properties of Alkali Hydrido-closo-borate Compounds
Source: Inorg Chem. 2022 Mar 24;61(13):5224–33. doi: 10.1021/acs.inorgchem.1c03681 (PMC8985130; doi:10.1021/acs.inorgchem.1c03681)
Supplement: Supplementary file 1 — ic1c03681_si_001.pdf [file ic1c03681_si_001.pdf]

SUPPORTING INFORMATION

Study of the temperature and pressure dependent structural properties of alkali hydrido-*closo*-borates compounds

Romain Moury[ab]*, Zbigniew Łodziana[c]*, Arndt Remhof[d], Léo Duchêne[da], Elsa Roedern[d], Angelina Gigante[ad] and Hans Hagemann[a]*

[a] Department of Physical Chemistry, University of Geneva, 30 Quai E. Ansermet, Geneva, 1211, Switzerland
[b] Institut des Molécules et Matériaux du Mans, University of le Mans, Avenue Olivier Messiaen, Le Mans, 72085, France
c Institute of Nuclear Physics, Polish Academy of Sciences, ul. Radzikowskiego 152, Kraków, 31342, Poland

[d] Empa, Swiss Federal Laboratories for Materials Science and Technology, Überlandstrasse 129, Dübendorf, 8600, Switzerland

*corresponding author: romain.moury@univ-lemans.fr, Zbigniew.Lodziana@ifj.edu.pl

Calculation procedure for the HT phase of $Li_2B_{10}H_{10}$.

More than 120 initial configurations were generated with Li^+ cations distributed at the tetrahedral (T) or octahedral (O) interstitial sites and $B_{10}H_{10}^{2-}$ anions with quasi-random orientations of the *ccp* packing of the cubic unit cell. There are 8 T sites and 4 O sites per conventional *ccp* unit cell - two cases were considered: all cations located at T sites and 50% of cations at O sites for the second case. For each of the interstitial site the cation was placed at its center and the anion orientations were generated randomly, including orientations along principal axis and lattice diagonals. The experimental unit cell with volume 866 Å^3 was used. The ground state energy for each configuration was obtained by relaxation of the internal degrees of freedom with conjugate gradient method. Following the optimization procedure the ground state energy was compared to the energy of the low temperature $P6_422$ hexagonal structure [1], and the distribution of these energies is depicted in Fig. 6. The calculated lattice parameters for LT structure are a = 7.0706 Å and c = 15.0046 Å.

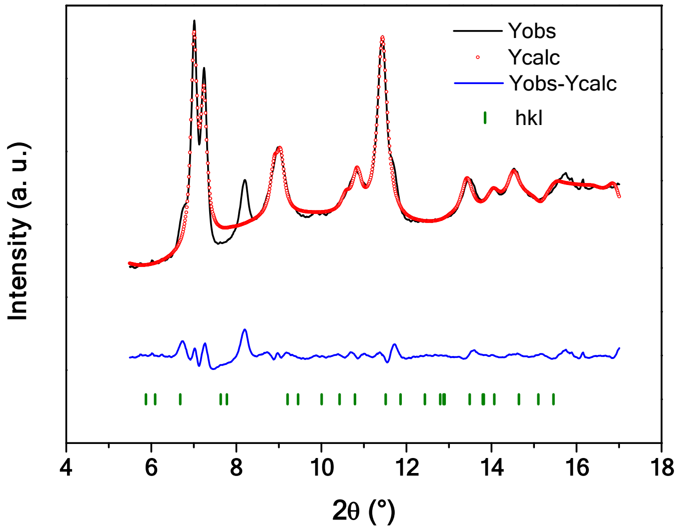

Figure S1. Rietveld refinement of hp-β-$K_2B_{12}H_{12}$ with *Pnnm* space group.

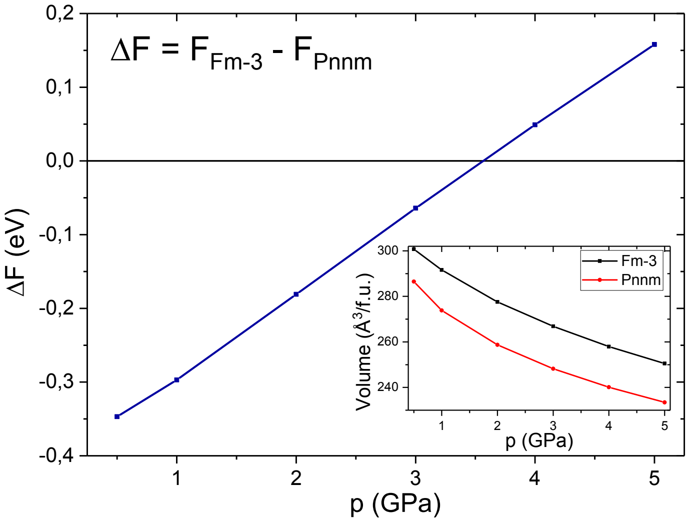

Figure S2. The calculated difference of the free energy between *Fm*-3 and P*nnm* structures of $K_2B_{12}H_{12}$ as a function of pressure. The insert shows specific volume for both phases.

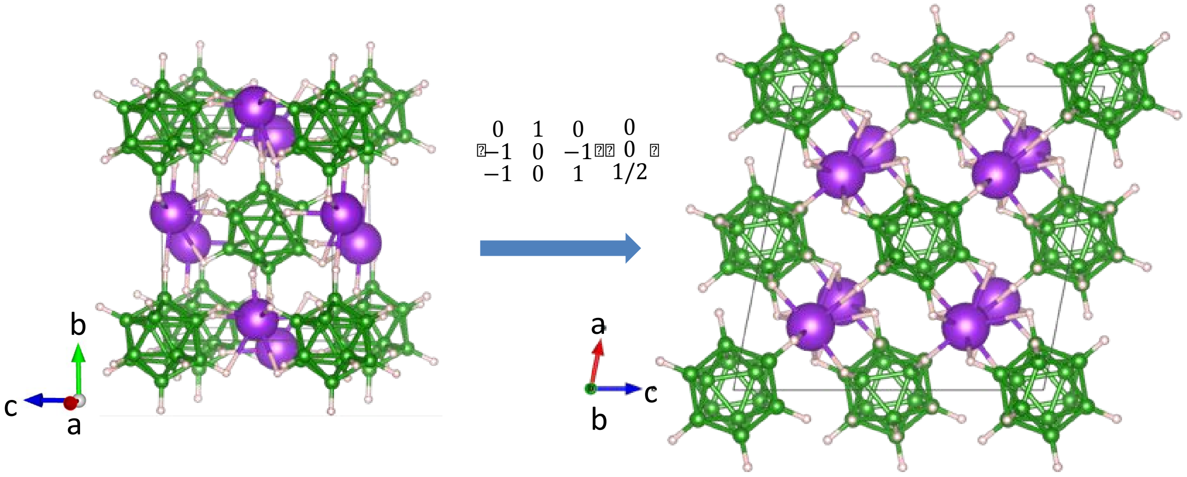

Figure S3. Transformation of hp-β-$K_2B_{12}H_{12}$ with *Pnnm* space group into $P2_1/c$.

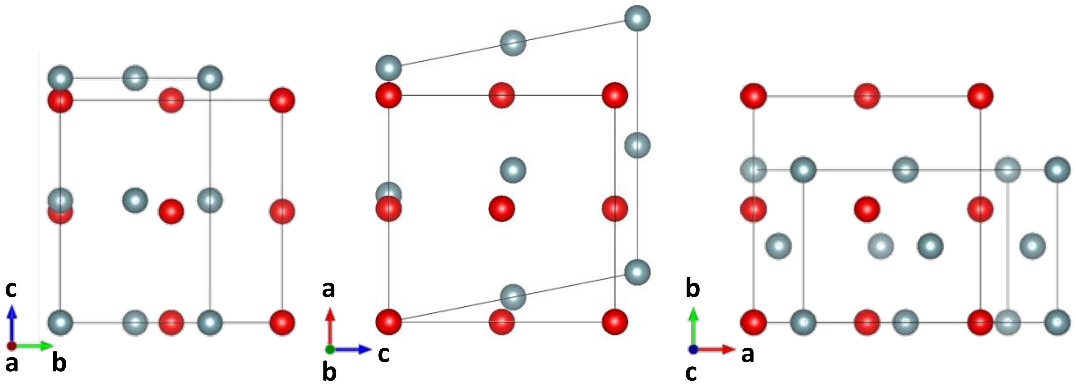

Figure S 4. Representation of the transformation of cubic lattice *Fm*-3*m* (for the sake of clarity $B_{12}H_{12}^{2-}$ units have been replaced by red balls) into the monoclinic lattice $P2_1/c$, (for the sake of clarity $B_{12}H_{12}^{2-}$ units have been replaced by green balls) along (a) [100] axis, (b) [010] axis and (c) [001] axis (lighter green balls correspond to the plan backward)

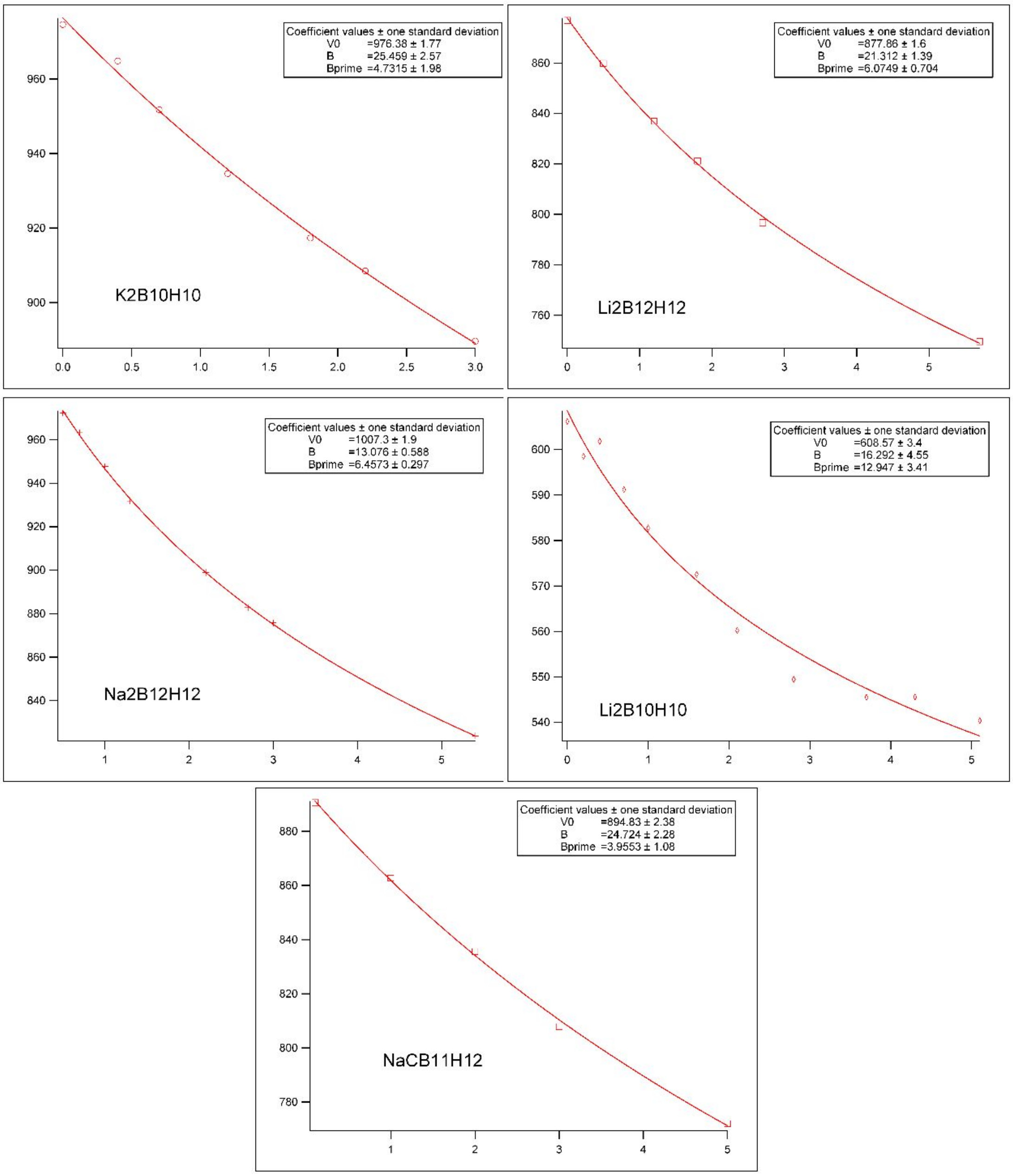

Figure S5. Variation of volume per formula unit as a function of pressure with the resulting fit using Murnaghan equation-of-state

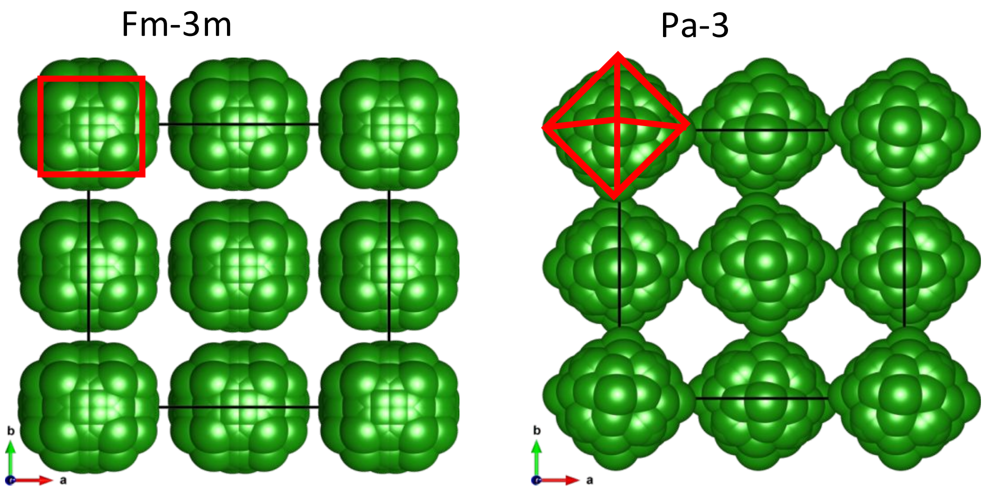

Figure S6. Oversimplified structures with *Fm*-3*m* and *Pa*-3 space groups highlighting the orientation of the $B_{10}H_{10}^{2-}$ ions. The red square indicate cubic shape for *Fm*-3*m* and octahedral one for *Pa*-3. The octahedral shape results from orientation of $B_{10}H_{10}^{2-}$ ions along three principal lattice axes. The anion alignment with four cube diagonals is required for Fm-3m symmetry.
